# Root Resection: Key Updates and a Review of the Literature

**DOI:** 10.7759/cureus.113050

**Published:** 2026-07-20

**Authors:** Jing V Sit, Mohammed I Khan, Hassan El-Awour

**Affiliations:** 1 Faculty of Dentistry, University of Toronto, Toronto, CAN; 2 Department of Oral and Maxillofacial Surgery, Faculty of Dentistry, University of Toronto, Toronto, CAN

**Keywords:** compromised tooth, furcation defect, furcation involvement, hemisection, periodontal surgery, root resection, tooth structure preservation

## Abstract

Root resective therapy remains a valuable but technique-sensitive treatment modality aimed at preserving multi-rooted teeth with severe periodontal or endodontic involvement. This review summarizes key updates in root resection, including its historical context, indications and contraindications, and evolving clinical approaches. Evidence demonstrates that both conventional root resection, often preceded by endodontic therapy, and vital root resection, performed prior to or without root canal treatment, can achieve favourable outcomes under specific conditions. Surgical techniques such as presurgical crown contouring and the vertical cut method offer distinct advantages, though direct comparative studies remain limited. Diagnostic challenges, such as assessing furcation involvement, have prompted refinement of classification systems and integration of advanced imaging modalities such as cone-beam computed tomography, which improve accuracy but carry higher costs and radiation exposure. Long-term survival rates of resected teeth vary widely, reflecting the importance of case selection, surgical proficiency, and supportive care. While extraction and implant placement are the most common treatments today, with the given indications, root resection provides the unique benefit of preserving natural dentition, underscoring its role as a last-resort but meaningful option in modern dentistry. Further standardized, long-term clinical trials are needed to clarify prognostic factors and optimize outcomes.

## Introduction and background

Root resective therapy is utilized as a last resort to save endodontically or periodontally compromised teeth. Primarily utilized in multi-rooted molars, these techniques involve the surgical removal of all or a portion of a tooth’s root [1]. Its predominant usage in molars is explained by the presence of the furcation, the anatomic region where roots diverge in multi-rooted teeth [1]. This region can be thought of as the space between the roots, normally filled by alveolar bone and tooth-anchoring periodontal structures. The process of furcation involvement, also known as furcation invasion, is the pathologic resorption of bone within the furcation caused by the extension of periodontitis or pulpitis into the furcation [1]. This process has multiple aetiologies with a well-studied relationship between that of the pulp and the periodontium [2-6]. Loss of attachment in the furcation exposes an area of roots where hygienic maintenance is hindered by the tooth’s own complex anatomy, often leading to further plaque buildup. Thus, an important goal of root resection is to improve accessibility to the furcation with the goal of making oral hygiene more manageable [2-4].

Although root resection has historically served as a valuable tooth-preserving treatment modality, its role in contemporary dentistry remains controversial. Advances in implant dentistry have shifted treatment paradigms toward extraction and implant replacement, while reported outcomes of root resection have varied considerably across studies [2,5]. Furthermore, successful treatment requires careful case selection, interdisciplinary management, and an understanding of the complex biologic relationship between pulpal and periodontal disease. As a result, uncertainty remains regarding the indications, contraindications, long-term prognosis, and appropriate clinical application of root resective procedures [2,5]. A thorough review of the available evidence is therefore warranted to clarify the current role of root resection in the management of compromised multi-rooted teeth and to assist clinicians in evidence-based treatment planning.

## Review

Methods

This article was designed as a narrative review to summarize key updates in root resective therapy, including its historical development, endodontic-periodontal considerations, furcation diagnosis, surgical approaches, survival outcomes, complications, and treatment alternatives. The aim of the review was to provide a clinically focused overview of root resection as a tooth-preservation strategy for multirooted teeth with advanced periodontal, endodontic, or combined involvement.

Relevant literature was identified through searches of PubMed/MEDLINE, Google Scholar, Scopus, Web of Science, ScienceDirect, and the Cochrane Library, as well as manual screening of reference lists from included articles. Search terms included combinations of “root resection,” “root amputation,” “hemisection,” “furcation involvement,” “furcation defect,” “endodontic-periodontal lesion,” “periodontal-endodontic lesion,” “vital root resection” with no restrictions on date, limited to articles published in English. Articles were excluded if they were not available in English, focused exclusively on single-rooted teeth, did not address root resection or its related clinical concepts, or lacked sufficient methodological or clinical detail to contribute meaningfully to the objectives of this review.

Studies were considered eligible for inclusion if they addressed root resection therapy, root amputation, hemisection, or related tooth-preserving surgical procedures in multi-rooted teeth. Original research studies, clinical trials, cohort studies, case series, case reports, systematic reviews, narrative reviews, and seminal historical publications relevant to the development and clinical application of root resection were included. Additional studies evaluating the biological basis of furcation involvement, endodontic-periodontal lesions, surgical techniques, restorative considerations, and long-term treatment outcomes were also considered.

As this manuscript is a narrative review rather than a systematic review or meta-analysis, no formal Preferred Reporting Items for Systematic Reviews and Meta-Analyses (PRISMA) screening process, risk-of-bias assessment, or pooled statistical analysis was performed. The included studies varied substantially in design, sample size, follow-up duration, treatment protocol, outcome definition, and complication reporting. Therefore, findings were synthesized narratively, and statistical values such as survival rates, P-values, hazard ratios, confidence intervals, and complication rates were reported only when provided by the original studies.

Pulp-periodontal complex

There are three major pathways that connect the pulp to the periodontium: the dentinal tubules, the apical foramen, and the lateral root canals [7]. It is common knowledge that pulpal infections can spread to periodontal structures through the apical foramen, ultimately destroying the surrounding periodontal structures [6,7]. This is the main pathway of communication between the pulp and the periodontium. This inflammatory process extends coronally until complete attachment loss occurs, resulting in the formation of a deep pocket [6,7]. Additionally, advancement of this inflammatory process towards lateral canals may lead to the impairment of pulpal blood supply from surrounding periodontal tissues, leading to pulpal necrosis [5,8]. Lateral canals, otherwise known as accessory canals, are small branches of the main root canals that exit laterally or coronally to the radiographic apex of the tooth and are concentrated towards the apical half of roots and in the furcation [1,4,5,8,9]. Through these lateral canals, lesions within the furcation can result in the spread of infection to the pulp, leading to necrosis [5,7,8,10]. Toxic products from endodontically compromised pulps can also tranverse through these same lateral canals to perpetuate periodontal lesions [8]. The pulp and the periodontium are a closely related complex; infection to one may eventually spread to the other.

Classification and clinical interpretation of endodontic-periodontic lesions

Lesions involving the pulp and periodontium may arise from different primary sources; classification is important when determining whether a tooth should be treated endodontically, periodontally, with resective therapy, or ultimately extracted [7]. Endodontic-periodontal lesions may be broadly categorized as primary endodontic lesions, primary periodontal lesions, primary endodontic lesions with secondary periodontal involvement, primary periodontal lesions with secondary endodontic involvement, or true combined lesions [7]. This distinction is clinically useful because lesions of primary endodontic origin may improve following root canal treatment (RCT), whereas lesions driven primarily by periodontal destruction, particularly when furcation involvement is advanced, may require periodontal therapy, regenerative or resective treatment, or extraction depending on the remaining support and restorability of the tooth [7].

Contemporary classification systems further emphasize prognosis-based decision-making in endodontic-periodontal lesions [11]. The 2017 World Workshop classification considers whether endodontic-periodontal lesions occur with or without root damage, such as fracture, perforation, or external root resorption, and whether the patient has periodontitis [11]. This distinction is clinically relevant because lesions associated with structural root damage generally carry a poorer prognosis, while lesions without root damage may be more amenable to combined endodontic and periodontal therapy when adequate support remains [11]. Therefore, assessment of endodontic-periodontal lesions should be considered alongside pulp vitality testing, probing depth patterns, furcation classification, radiographic or cone beam computed tomography (CBCT) findings, remaining bone support, and restorability when deciding whether root resection is appropriate [7].

Endodontics and root resection

Due to the relationship between the pulp and the periodontium, furcation involvement is often concomitant with pulpal involvement [9]. Regardless of its origin, the treatment of a furcation defect often involves a combined endodontic-periodontal approach. In fact, studies suggest endodontic therapy is necessary before the healing of periodontal tissues can occur [5,6,10]. Inversely, root canal therapy alone has not been proven to resolve advanced furcation involvement in maxillary molars [2,5,12]. Failure to treat both the pulpal and periodontal tissues often results in poor prognosis and subsequent extraction of the tooth [5].

RCT is strongly recommended prior to resecting roots [2,4,13,14]. Initiating with endodontic treatment provides the clinician with information about the tooth’s internal root morphology. In some cases, RCT cannot be completed due to the obstruction of the canal by calcification or dentin deposition. If root resection was initiated prior to RCT and then the tooth is unable to be endodontically treated, due to obstruction or complications, like vertical root fracture, patients may be displeased with their painstaking time and financial investment. Filling of the root canals with amalgam or gutta percha also provides an important visual landmark during the resective procedure [3]. By starting with RCT, clinicians can sequentially move forward in the event of a successful endodontic prognosis.

The benefits of conventional root resection with endodontic therapy first include providing information on the internal root canal morphology of retained roots; filling the root with foreign materials may act as a useful visual landmark in root resective procedures; and the prognosis of endodontic treatment and the presence of endodontic complications can significantly alter the overall treatment plan so performing it first provides more information on the predictability of treatment approaches and outcomes [2-7].

Despite this, past studies have demonstrated that vital root resection, the removal of a root prior to endodontic treatment, is a viable option to conducting root resection treatment [2,13]. In a clinical and histological study of 26 teeth, Smukler & Tagger (1976) utilized vital root resection two weeks prior to endodontic treatment with success [13]. Although Smukler & Tagger (1976) definitively concluded that endodontics prior to root amputation should remain the treatment of choice, they found that vital root resection could still produce good results without significant endodontic and postoperative sequelae [13].

In a more recent study, Sanz et al. (2020) introduced a novel method of vital root resection in which deep pulpotomy was performed a month prior to root resection therapy [15]. In that study, 15 maxillary molars with advanced furcation defects and vertical bone loss were resected utilizing the presurgical crown- contouring method and followed up for seven years [2]. Jepsen et al. (2020) did not utilize root canal therapy at any point in their treatment plan, opting to retain pulp vitality in retained roots [2].

More recent case-series evidence has continued to investigate whether vital root resection can be performed in a more conservative manner while preserving the vitality of the retained roots. Ciardo et al. (2024) described vital root resection with radicular retrograde partial pulpotomy in furcation-involved maxillary molars affected by periodontitis [16]. In that case series, seven vital maxillary molars with residual probing pocket depths (PPDs) of at least 6 mm and class II or greater furcation involvement were treated using mineral trioxide aggregate, and all seven molars remained in situ at a mean follow-up of 26.84 ± 5.37 months.

Similarly, Chen et al. (2025) described a single-visit vital root resection approach combined with retrograde pulpotomy for periodontally diseased molars, with follow-up extending up to three years [17]. This approach differs from conventional root resection because it attempts to preserve pulp vitality in the retained roots while surgically managing the diseased or periodontally compromised root. Watanabe et al. (2025) also reported vital root resection for the management of furcation involvement in a mandibular molar with radix entomolaris, further demonstrating the adaptability of this approach in anatomically complex cases [18].

The advantages of vital root resection include the potential to avoid endodontic complications, costs, and loss of tooth structure [2]. Avoiding root canal treatment demonstrates a 75% decrease in both cost and treatment time [2] and allows the operator to affirm the clinical status of the furcation, which is often difficult to accurately diagnose [19]. Despite this, the contamination of the pulp is a potential downside to this form of treatment, which could be detrimental in the long term should infections spread to the periodontium [2].

Current evidence on vital root resection remains limited mainly to case series and case reports; vital root resection should be interpreted as a promising but still developing treatment approach rather than a predictable replacement for conventional root resection.

Root resection techniques

Root resection has a variety of names and is often used interchangeably with terms such as radectomy, radisection, root amputation, hemisection, bicuspidization, and root separation. This lack of uniformity in terminology has been noted in past literature [10]. In recent times, and in accordance with the American Academy of Periodontology, root resection has been commonly referred to as the removal of all or a portion of a tooth’s root and it is performed in one of two ways: root amputation or hemisection [1,20]. Root amputation involves the removal of one or two roots from the tooth in a manner in which the overlying crown is retained [21,22]. Hemisections, which are generally done in mandibular molars, involve the separation of a multirooted tooth through the furcation in a way that the resected portion of the root can be extracted with its associated crown or restored [1]. Details of these procedures were thoroughly described by Kirchoff & Gerstein’s (1969) presurgical crown-contouring method and Weine’s (2004) vertical cut method [3,4].

**Figure 1:**
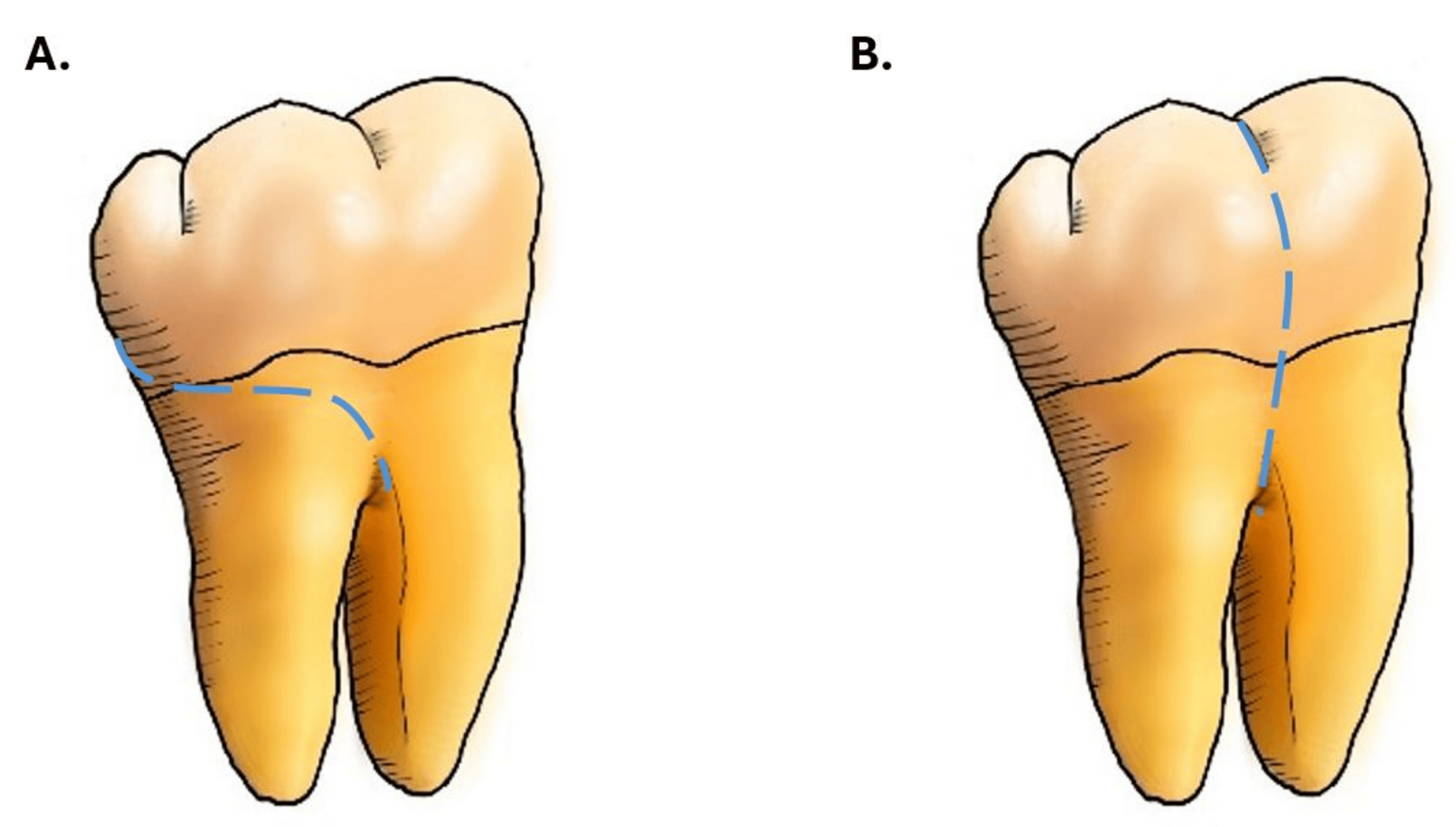
Illustration of Kirchoff & Gerstein's presurgical crown-contouring method (A) and Weine’s vertical cut method (B) The figure is an original artwork created by Jing Vincent Sit using the Procreate app on an iPad.

The presurgical crown-contouring method, visualized in Figure 1A, involves the reduction of a portion of the crown overlying a soon-to-be amputated root [3,4]. This method of root resection is especially effective for maxillary molars with periodontal defects as the bone loss surrounding a root allows superior access to the cementoenamel junction, the landmark at which this preparation is initiated [3,4]. The trimming of the crown is aided by the use of amalgam or gutta percha as a canal filling material, another important landmark in this procedure [3,4]. Through the use of a tapered fissure bur, the crown is contoured towards the furcation until root separation occurs [3,4]. The root can then be extracted before final restorative procedures are done. If the tooth is not to be used for prosthetic purposes with no intention of crowning, restorative procedures should be done to direct occlusal forces along the long axis of the tooth and to smoothen the tooth to facilitate proper oral hygiene [3]. Based on the retainment of a portion of the overlying crown, this method of root resection is utilized in root amputation procedures.

**Figure 2:**
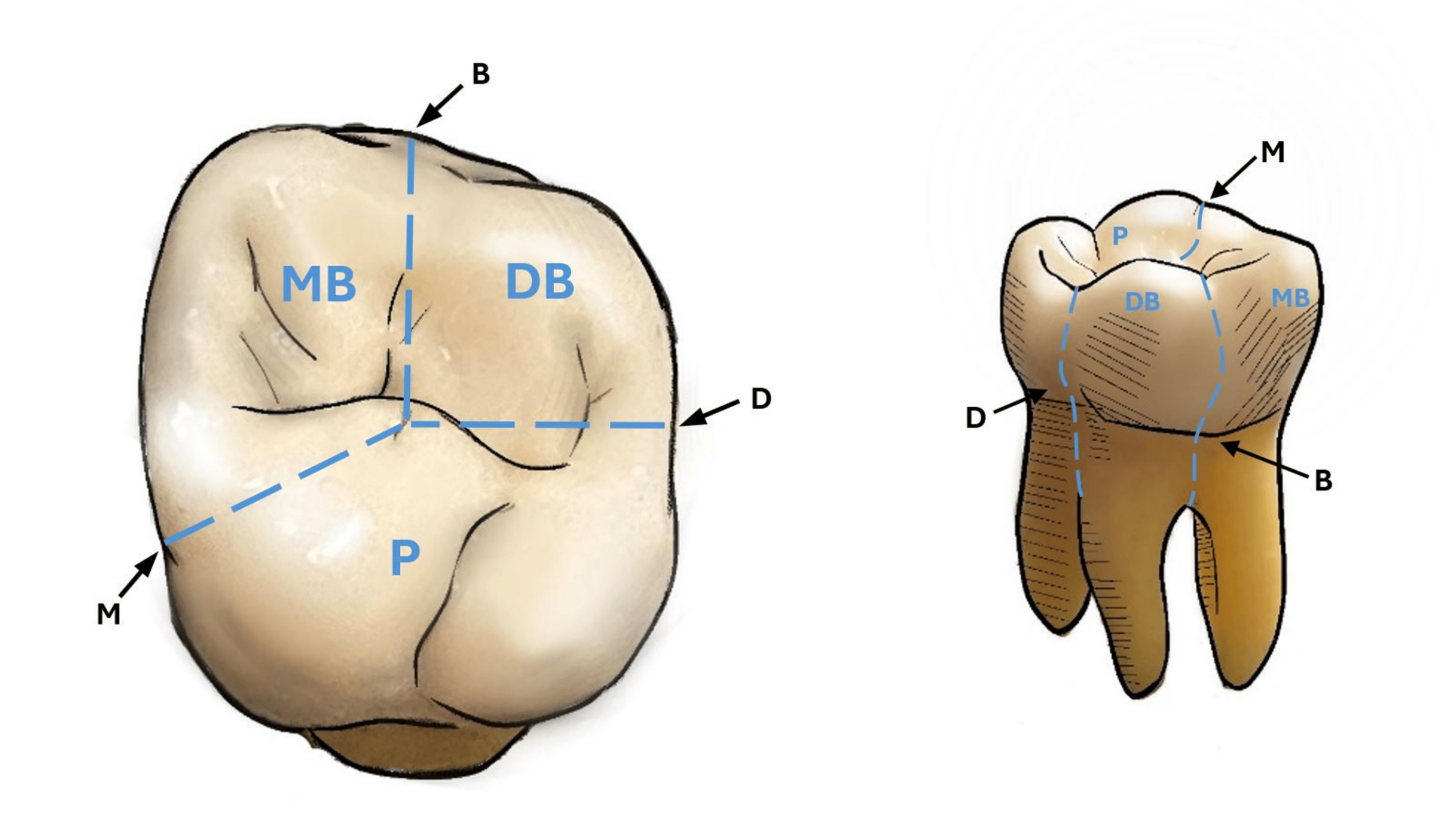
Guide for usage of the vertical cut method in maxillary molars with labelled furcation entrances The figure is an original artwork created by Jing Vincent Sit using the Procreate app on an iPad. MB: Mesiobuccal; DB: Distobuccal; P: Palatal; M: Mesial; D: Distal

The vertical cut method, as the name suggests, involves the usage of a long shank, tapered fissure bur to section through the entire crown to the furca in a vertical motion [4]. In doing so, the entire root and its overlying crown can be removed [4]. This process is visualized in Figure 1B. This method differs between maxillary and mandibular molars due to their respective anatomy. In mandibular molars, where there are two roots, the resectioning of the tooth can be accomplished in a straight cut to split the tooth in half. This cut connects the two furcation entrances, located on the buccal and lingual aspects of mandibular molars, to produce two separate bicuspid-like tooth structures. Note that in a bicuspidization procedure, both bicuspids would be restored and retained [4]. However, in a hemisectioning procedure, one of the bicuspids is removed while the other is restored and retained [4]. This procedure becomes slightly more complex in maxillary molars due to the presence of three roots, resulting in three furcation entrances. The three roots, which are found mesiobuccally, distobuccally, and palatally, produce three furcation entrances on the buccal, mesial, and distal aspects of maxillary molars. The mesial entrance is located two-thirds of the way interproximally towards the palatal surface, while the distal entrance is found directly under the interproximal contact at the halfway point [14,15,23,24]. As such, resectioning of the tooth must be done in a way that matches the anatomy of the furcation; this is visualized in Figure 2.

Both methods of conducting root resection have been successfully utilized in various published case reports [2,3,21,23,25]. The presurgical crown-contouring method is a logical approach to root resective therapy in that it conserves as much tooth structure as possible in the process of resolving furcation defects. However, there are two major pitfalls for the usage of this method of root resection in comparison to the vertical cut method [4]. The vertical cut method will always produce a more accessible, exposed furcation due to the complete removal of the anatomical crown that accompanies the amputated root [4]. If the goal of root resection was to eliminate a furcation defect and to increase accessibility for the purpose of hygiene maintenance, the vertical method is the clear choice. The conserved crown produced from a presurgical crown-contouring procedure would also dissipate occlusal forces in an undesirable manner, assuming normal occlusion is maintained [4]. Since only the underlying root is amputated, the clinician will need to adjust the occlusion in a manner in which forces are going to dissipate along the long axis of the remaining roots.

The advantages of the vertical cut method include direct visualization of the bur penetration, to ensure the preparation is in the correct position; removal of the portion of the crown over the resected root structure, which may prevent undesirable occlusal forces; and the position of each cut, based on the anatomy of the furca, allows the root to cleave along desirable angles and contours; and the technique provides excellent visualization of the furca following amputation allowing for easier trimming or smoothing of sharp edges [12,14,20].

In contrast, the presurgical crown-contouring method is advantageous in that it simplifies the amputation procedure; aids in prolonging retention and utilization of a tooth; less debris enters an exposed socket, since root extraction occurs after crown contouring; and it conserves the anatomical crown, resulting in superior aesthetics and contacts [3,11,16].

While the vertical cut method is superior in theory, there are no studies that formally evaluate the efficacy of both methods in relation to one another. Past literature has also demonstrated the flexibility of root resective therapy as they have been modified to meet the demands of a particular case with respect to vital root resection procedures, intentional replantation procedures, and multi-rooted maxillary premolars among other examples [2,13,22,26].

Furcation involvement classification

Much like the terminology related to root resection, the classification of furcation involvement has been revised repeatedly over the course of the last century with little standardization between reports. This is because authors often recognized limitations in previous classification systems and attempted to create a more comprehensive system. Classification systems allow clinicians to characterize and interpret the severity of furcation defects, with higher grades often indicating a need for root resection therapy. Early furcation classification systems were designed to describe the main characteristics of the furcation defect as well as the horizontal or vertical attachment loss [27,28]. Later classification systems were designed to provide a more complete picture by accounting for remaining bony walls, morphology of existing bone, and the relationship between root trunk and attachment loss [27].

The preliminary classification systems, found in Table 1, are often found in the literature and act as the basis for many of the classification systems proposed by independent authors. Glickman (1953) is often credited with being one of the first to characterize furcation defects, and as such, presents the most commonly utilized classification system. However, Glickman’s (1953) classification system has several limitations, namely, it lacks numerical values and involves subjectivity in its criteria, limiting its effectiveness in characterizing furcation defects reliably. While Hamp et al. (1975) proposed a more precise classification system with numerical values, their system had trouble differentiating instances in which the horizontal loss of tissue was equal to 3 mm [27,29].

**Table 1:** Classification systems for furcation involvement *NE: Not exposed; E: Exposed furcation

Author(s)	Year	Classification System
Glickman, I. [28]	1953	Grade I – early or incipient lesion. Grade II – loss of interradicular bone and pocket formation, but a portion of alveolar bone and PDL is intact. Grade III – through-and-through lesion. Grade IV – through-and-through lesion with gingival recession leading to a clearly visible furcation area
Hamp et al. [29]	1975	Degree I – horizontal loss of periodontal tissue support less than 3 mm. Degree II – horizontal loss of support exceeding 3 mm, but not encompassing the total width of the furcation. Degree III – horizontal “through-and-through” destruction of periodontal tissue in the furcation
Ramfjord & Ash [30]	1979	Class I – tissue destruction <2 mm (<1/3 of tooth width). Class II – tissue destruction >2 mm (>1/3 of tooth width). Class III – through-and-through involvement
Walter et al. [31]	2009	Degree 0 – furcation not accessible with a periodontal probe. Degree I – horizontal loss of periodontal tissue support up to 3mm. Degree II – horizontal loss of support exceeding 3 mm, but no more than 6mm. Degree II–III – horizontal loss of support exceeding 6 mm, but no detectable ‘‘through and through’’ destruction. Degree III – horizontal ‘‘through and through’’ destruction of the periodontal tissue in the furcation
Pilloni & Rojas [27]	2018	NE I – furcation lesion not clinically exposed, horizontal attachment loss is 2 mm or less. NE II – furcation lesion not clinically exposed, horizontal attachment loss is 3 mm or more. NE III – furcation lesion not clinically exposed, horizontal attachment loss is total with through and through opening of the furcation. E I – furcation lesion clinically exposed, horizontal attachment loss is 2 mm or less. E II – furcation lesion clinically exposed, horizontal attachment loss is 3 mm or more. E III – furcation lesion clinically exposed, horizontal attachment loss is total with through and through opening of the furcation

In a review study of past furcation classification systems, Pilloni & Rojas (2018) proposed a novel system that segregates furcation defects based on the presence (E) or absence (NE) of an exposed furcation lesion [27]. This change addresses a limitation in Glickman’s (1953) classification system in that gingival recession, which clinically exposes the furcation, is addressed in Grade IV but not the other grades. In the event of gingival recession occurring independently of furcation involvement, this change can provide a more complete picture of each case.

Indications for root resective therapy were described by Weine (2004) as either endodontic/restorative or periodontal. The endodontic/restorative indications include prosthetic failure of piers or abutments within a splint; endodontic failure; vertical fracture in one root; and severe destructive process that makes a portion of a multirooted tooth unrestorable [4]. The periodontal indications include severe vertical bone loss involving one root of a multirooted tooth; through-and-through furcation destruction that may not be corrected by periodontal surgery; unfavourable proximity of roots of adjacent teeth, preventing adequate hygiene in maintenance of proximal areas; and severe root exposure due to dehiscence [4]. Other indications for root resective therapy include class II or III furcation involvement; root caries limited to one root; endodontic treatment required but a root canal is inaccessible; periapical osteitis, root cysts, root resorption, and obliteration of root canal; loss of root substance; marginal periodontal disease; mandibular fracture or operation to correct prognathism; and horizontal fracture in one root [3,20,25,32,33].

Weine’s (2004) contraindications for root resective therapy include strong adjacent teeth available for bridge abutments; inoperable canals in retained root(s); and root fusion with separation impossible. Other contraindications include closely approximated roots; inadequate bone support on remaining roots or unfavourable anatomical factors; significant discrepancies in adjacent interproximal bone height; and remaining roots that cannot be restored/endodontically treated [20,32].

Diagnosis of furcation involvement

Furcation involvement is primarily determined through clinical and radiographic evidence and is a direct consequence of the progression of periodontal disease. From a clinician’s perspective, the diagnosis of furcation involvement may appear to be a straightforward process. However, past studies by Zappa et al. (1993) have concluded that clinical measurements had limited value, displaying a difference in measurement of up to 9 mm when compared to measurements derived from surgical exposure [19].

In this study, dentists classified the furcations using the Ramfjord index as well as the Hamp index [19]. By surgically exposing the furcations and using silicone as an impression material, accurate measurements were made to see how effective clinicians were when it came to diagnosing the extent of furcation defects [19]. This study highlighted the fact that not only were clinical measurements inaccurate, the classification system utilized also had a role in this inaccuracy [19]. Readings made with the Ramfjord index resulted in overestimations in degree 1 (5%), degree 2 (40%), and degree 3 (43%) furcation-involved molars [19]. In comparison, readings made with the Hamp index resulted in overestimations in degree 1 (7%) and degree 2 (24%) but not in degree 3 (0%) furcation-involved molars [19]. Failure to adequately diagnose a furcation defect may result in over or undertreatment.

These inaccuracies are explained by the limited dimensional perspective provided by radiographs, the lack of direct visibility of the furcation, and the poor accessibility of conventional periodontal probes and curettes. In fact, in a previous study of 114 maxillary first molars and 103 mandibular first molars, Bower (1979) concluded that conventional curettes are ineffective when it comes to accessing and maintaining furcations [34]. The curette, being the instrument of choice to remove plaque and calculus on roots, has a blade face width ranging between 0.75 mm and 1.10 mm, while 81% of furca entrances had a diameter of 1.0 mm or less and 58% of furca entrances had a diameter of 0.75 mm or less [34]. This implies that curettes will be ineffective in accessing and maintaining plaque buildup in more than half of all furcation-involved first molars.

Various studies have been undertaken with the goal of improving the standards for clinical furcation diagnosis. In 1986, Hardekopf et al. discussed at length and coined the term “furcation arrow”, which describes the presence of a small, triangular radiographic shadow that commonly overlies mesial or distal roots in furcation-involved maxillary molars [35]. In that study, approximately 150 maxillary molars with varying degrees of furcation involvement were assessed visually. In comparison to 120 maxillary molars that acted as controls, Hardekopf et al. (1986) believed that the association between the furcation arrow and degrees 2 and 3 furcation involvement was significant, as the radiographic shadow was rare in uninvolved furcations [35]. However, studies have questioned the clinical reliability of the furcation arrow, stating that it was only accurate in correctly predicting furcation involvement 72% of the time when present on radiographs while also only being present 39% of the time when furcation involvement was surgically confirmed [36]. It is questionable if furcation arrows can be used as a genuine diagnostic marker.

Bone sounding of the interradicular bone has been cited to improve diagnostic accuracy [37]. One study measured and compared vertical and horizontal depths of 274 furcations at three separate time points: periodontal probing before local anesthesia, bone sounding after local anesthesia, and by direct measurement during surgery [37]. In that study, it was found that traditional methods of periodontal probing yielded significant underestimations of 1.80 mm vertical and 2.16 mm horizontal depths in comparison to the surgically measured depths of 2.79 mm and 3.65 mm, respectively [37]. When local anesthesia and bone sounding were utilized, the measurements became much closer at 2.40 mm and 3.11 mm, respectively (P = 0.000) [37].

Additionally, advances in medical imaging technology can play a role in improving diagnostic accuracy for furcation defects. The application of CBCT systems, three-dimensional imaging technology, in the field of periodontics has shown promise in recent times. In a clinical study of 12 patients with generalized chronic periodontitis, furcation defects in 22 maxillary molars were analyzed with traditional methodologies as well as CBCT [31].

It was found that the degree of furcation involvement was accurate in 27%, overestimated in 29%, and underestimated in 44% of sites [31]. Additionally, using the modified version of the Hamp index, degree I furcations were found to be underestimated 25% of the time, degrees II and II-III furcations were underestimated up to 75% of the time, and degree III furcations had no discernible difference when diagnosis was done traditionally or with CBCT [31]. Through these findings, it was concluded that CBCT images of maxillary molars were a reliable basis for treatment planning [31]. However, while the superior accuracy provided is indisputable, the accessibility of CBCT technology in private practices, the increased accompanying costs, as well as the increased dosage of irradiation when compared to conventional periapical radiographs necessitate consideration for both clinicians and patients [31,38].

More recent literature continues to support the use of CBCT as an adjunctive diagnostic tool for furcation assessment, particularly when clinical probing and two-dimensional radiographs do not provide sufficient information for treatment planning. In a systematic review evaluating diagnostic imaging methods for furcation involvement, Jolivet et al. (2022) emphasized that accurate diagnosis is essential because the prognosis and treatment strategy for multirooted teeth are strongly influenced by the degree of furcation involvement [39].

Similarly, Alotaibi et al. (2024) found that CBCT was a reliable adjunctive method for detecting furcation involvement in maxillary teeth and reported that clinical examination alone may not be sufficient in all cases [40]. These findings are consistent with earlier evidence suggesting that CBCT can improve visualization of furcation morphology by providing three-dimensional assessment of interradicular bone loss.

However, CBCT should remain selectively indicated rather than routinely used, given its higher cost and radiation exposure compared with conventional radiographs. Therefore, CBCT may be most appropriate in advanced class II or class III furcation involvement, maxillary molars with complex anatomy, unclear clinical or radiographic findings, or cases where imaging results may directly influence whether the clinician chooses regeneration, root resection, or extraction.

Common clinical signs of furcation involvement include inflammation or bleeding on probing, deep pockets, gingival recession, tooth mobility, and communication between furca entrances [19,27,35,38]. Radiographic signs can include radiolucency around one root, radiolucency of the interradicular bone, and the presence of a furcation arrow [19,27,34,38].

Endodontic-related complications

While root resective therapy is often done to accommodate and treat furcation defects, oftentimes clinicians will need to employ these procedures when faced with endodontic complications. Endodontic complications, such as an obstructed, perforated root canal or a root fracture, would normally necessitate extraction. However, in some cases, resection of an undesirable root can potentially spare the remaining tooth regardless of the status of the furcation [41-43].

Anatomical challenges

The anatomical differences between maxillary and mandibular molars necessitate clinical considerations when furcation involvement is present. Some morphological factors that contribute to the etiology and prognosis of furcation-involved teeth include the number of roots, location and width of furcation entrance, root concavities, ridges, enamel pearls, cervical enamel projections, and root trunk length [20]. Cervical enamel projections in particular are correlated with furcation involvement as Masters and Hoskins (1964) noted their presence on 90% of furcation-involved mandibular molars while Hou and Tsai (1986) noted their presence in 82.5% of furcation-involved molars yet only in 17.5% of molars without furcation involvement [44,45]. Due to various anatomical features, there are key differences between maxillary and mandibular molars when it comes to the diagnosis, treatment, and prognosis for root resective treatment.

Maxillary molars are the most anatomically complex teeth due to the presence of the palatal root. The additional root introduces another dimension to the furcation as well as another opportunity for endodontic complications. Maxillary molars are also more prone to furcation involvement than mandibular molars [46]. In the context of root resective treatment, the additional root is detrimental, and the overall prognosis of class II or III furcation-involved maxillary molars is commonly considered to be hopeless [2-4].

In maxillary first molars, the distobuccal root is the root that is most frequently resected as well as the root with the most favourable prognosis when removed [4]. This is because the distal aspect of the maxillary first molar is prone to periodontal breakdown whilst the distobuccal root simultaneously provides the lowest area of attachment amongst the three roots [4,20,24]. It is believed that the close proximity of the distobuccal root of the maxillary first molar to the mesiobuccal root of the maxillary second molar contributes to periodontal breakdown [24]. In addition, and as discussed prior, relative to the palatally displaced mesial furca entrance, the interproximally located distal furca entrance is more challenging to maintain, making it more prone to decay.

The mesiobuccal and palatal roots of the maxillary molar are superior to the distobuccal root in their ability to resist mechanical stresses as well as their surface area for attachment [4,20,47]. In a past study where 20 maxillary first molars were cross-sectioned every millimetre to calculate the average area of available attachment, it was noted that there was no significant difference between the mesiobuccal and palatal roots, while the distobuccal root had significantly less area of available attachment [47]. In a separate study of 114 maxillary first molars, it was noted that 94% of mesiobuccal roots, 31% of distobuccal roots, and 17% of palatal roots were concaved on the furcal aspect [48]. While the palatal root was clinically longer and larger than the mesiobuccal root, the concavity and extension of the mesiobuccal root in the buccopalatal dimension provide it with a similar surface area [47]. The under or overestimation of the mesiobuccal or palatal roots due to this extension in dimension is a common cause of errors during root resective procedures [4]. Due to the superior retentive properties of the mesiobuccal and palatal roots, splinting is required if either root is resected [4]. The redistribution of occlusal forces should also be considered if the palatal root is resected.

Maxillary second molars pose a larger challenge in terms of root resective procedures as they are positioned more posterior, have a more apically positioned furcation, closer approximated roots, and more variations in size compared to maxillary first molars [4]. Moreover, the palatal root of maxillary second molars is prone to fusing to either buccal root in 10% of cases [4].

Mandibular molars, while less anatomically complex than maxillary molars, do present some challenges. These molars conventionally have two roots of equal length with the mesial root being wider buccolingually [4]. In a study of 103 mandibular first molars, concavities were found on the root surfaces of 100% of mesial roots and 99% of distal roots [48]. The mesial root of mandibular molars tends to have concavities on both mesial and distal aspects of the root, providing a figure-eight shape when cross-sectioned [4]. This figure-eight shape provides the mesial root with superior retentive properties in comparison to the distal root, which tends to have just one concavity. When resistance to mechanical stresses is prioritized, it may be beneficial to retain the mesial root over the distal root. However, the common occurrence of two root canals within the mesial root brings on greater potential for endodontic complications [4].

The root trunk is shorter on the buccal aspect compared to the lingual aspect on both mandibular first and second molars, making the tooth more prone to furcation involvement on the buccal aspect whilst also being easier to maintain [15]. Similar to maxillary second molars, mandibular second molars demonstrate a trend of being more difficult to manage with respect to root resective treatment. They have more variations in size and shape, a more apically situated furcation, and closer approximated roots in comparison to maxillary first molars [4].

Root resective procedure steps

**Figure 3:**
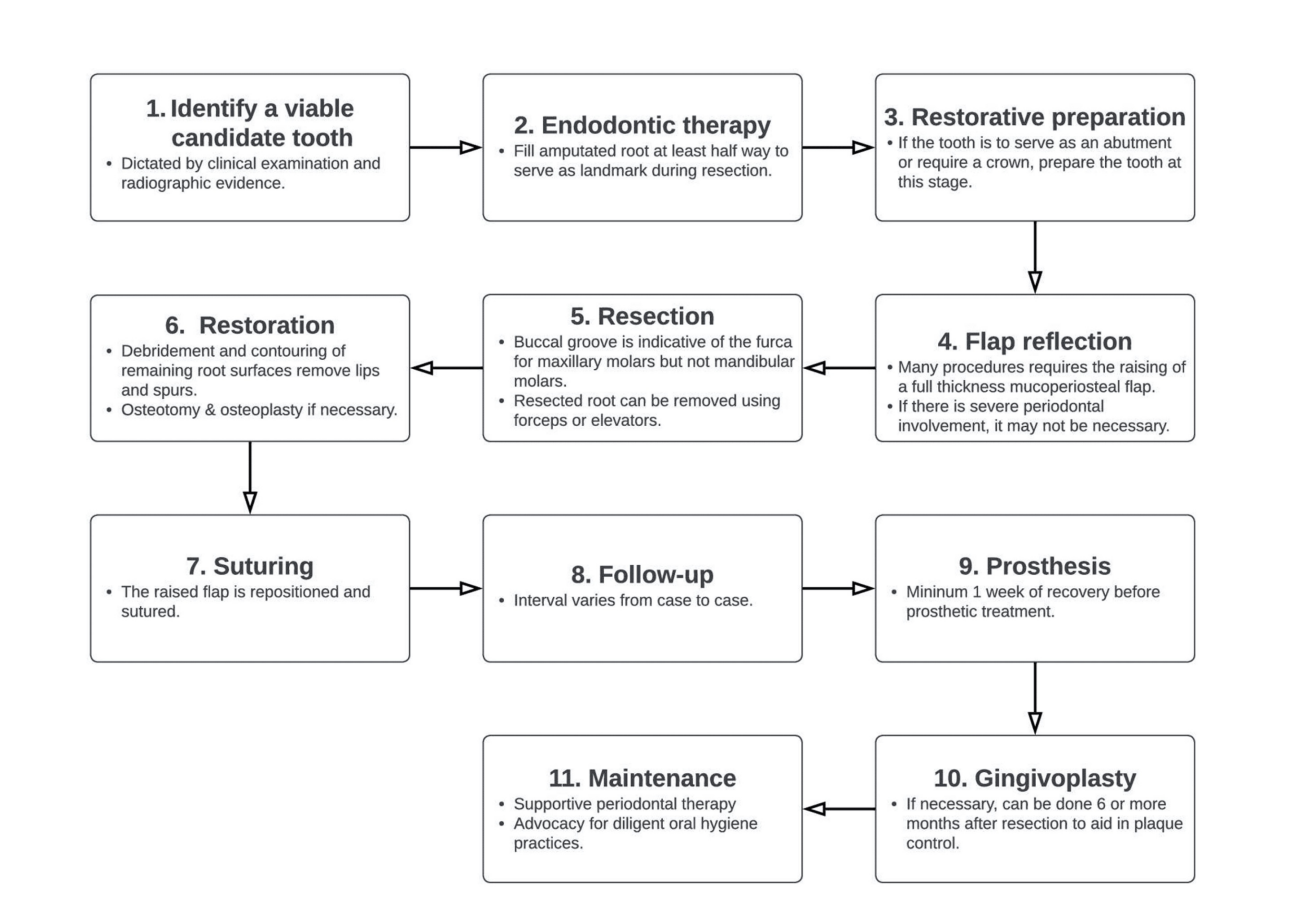
Algorithmic flowchart to conducting root resective therapy

The conventional method of conducting root resective therapy is diagrammed in Figure 3. This flowchart was designed based on stepwise advice provided by Bergenholtz (1972) and Weine (2004) [4,33]. The flowchart features a conventional methodology to conducting root resective therapy in which endodontic therapy precedes periodontal therapy. Most steps are completed under local anesthesia, and it is common for endodontic treatment, restorative treatment, and periodontal treatment to be done on different days. Due to the various skills demanded by the clinician to complete this treatment plan, the procedure is highly technique sensitive. Failure at any stage of the treatment will negatively impact sequential steps. Gingivoplasty, while not discussed by Bergenholtz (1972) or Weine (2004), has been cited in past literature as a potential final step in which the clinician is required to aid in creating a more suitable environment for plaque control [5]. Aside from surgical technique, case selection is crucial to successful root resective therapy [5,21,22,32,49].

Survival rates

There have been many long-term retrospective studies on the efficacy of root resective therapy, documenting survival rates as well as their complications (see Table 2 for details). Survival rates ranged from 18.4 to 100% with follow-up periods ranging from under one year to over twenty years. Based on these studies, it can be concluded that root resection, with appropriate case selection, is a viable and moderately predictable procedure. Although it is time- and resource-consuming, its value is in its ability to retain teeth that were previously indicated for extraction. The primary complications that arose were of periodontal, endodontic, root fracture, and carious origin. Although other causes of failure occurred, they were rarely reported or elaborated on. Successful treatment of a tooth has been found to restore tooth functionality by resolving furcation defects, preventing further gingival recession, reduce tooth mobility and pocket depths [5,25,50-57]. Recent evidence continues to support root resection as a selective tooth-preservation strategy, although survival remains dependent on case selection, periodontal stability, restorative design, and maintenance. Oh et al. (2025) evaluated root-resected molars over 1 to 13.4 years and reported that 21 of 60 teeth were extracted, corresponding to a 35% failure rate. Factors such as initial diagnosis, complete-coverage crown status, and tooth location were associated with time to tooth loss [58].

**Table 2:** Long-term studies on root resection, their survival rates, and complications y: years; mx: maxillary; md: mandibular; PPD: probing pocket depth; MTA: mineral trioxide aggregate

Author(s)	Publication Year	# of Patients	# of Teeth	Teeth Composition	Survival Rate	Follow-up Period	Notes	Complications					
								Periodontal	Endodontic	Root Fracture	Caries	Other	Total Failures
Bergenholtz [33]	1972	40	45	Mx. teeth (15) Md. teeth (30)	93.3%	11y	-	2 (66.7%)	1 (33.3%)	0 (0%)	0 (0%)	0 (0%)	3
Klavan [49]	1975	29	34	Mx first molar (30) Mx second molar (4)	97%	3y (mean) 1-7 years	One tooth was extracted and three developed mobility.	Not specified					1
Hamp et al. [29]	1975	-	87	Mx. first molar (28) Mx. second molar (11) Mx. first premolar (3) Md. first molar (30) Md. second molar (14) Md. third molar (1) Other premolars (1)	100%	5y	-	0 (0%)	0 (0%)	0 (0%)	0 (0%)	0 (0%)	0
Langer et al. [55]	1981	100	100	Md. molars (50) Mx. molars (50)	62%	10y	15.8% of failures occurred within five years. 55.3% of failures occurred between years five and seven. Most periodontal complications were found in Mx. molars, though there were two times greater overall failure in Md. molars.	10 (26.3%) Mx. molars more common 7:3.	7 (18.4%) Md. molars more common 4:3.	18 (47.4%) Md. molars more common 15:3.	3 (7.9%) Md. molars more common 3:0.	0 (0%)	38
Erpenstein [51]	1983	28	34	Mx. first molar (19) Mx. second molar (8) Md. first molar (6) Md. second molar (1)	79.4%	2.9y (mean) 1-7y	Significant reduction in periodontal pocket depths. Notably higher rates of endodontic failure compared to periodontal failure. Most mandibular molars served as abutments.	1 (14.3%)	6 (85.7%)	0 (0%)	0 (0%)	0 (0%)	7
Buhler [50]	1988	17	28	Mx. first molar (12) Mx. second molar (4) Mx. premolar (1) Md. first molar (9) Md. second molar (4) Md. third molar (1) *One patient dropped out, 3 resection cases lost.	68%	10y	No failures four years out. 32.1% failure 8-10 years out. At five years, 18/31 teeth were free of periodontal pockets. Most roots displayed loss of alveolar bone after 10 years, though it was not uniformly lost across all patients.	2 (22.2%)	3 (33.3%)	1 (11.1%)	0 (0%)	3 (33.3%) *2 combined periodontal-endodontic failures & 1 loss of retention.	9
Carnevale et al. [57]	1998	72	175	Mx. & Md. first molars (110) Mx. & Md. second molars (61) Mx. & Md. third molars (2) Mx. first premolars (2)	93%	10y	At the 10-year mark, the survival rate was 93% compared to 99% in control sites. Control sites represented teeth in patients that did not undergo root resection.	3 (25.0%)	4 (33.3%)	2 (16.7%)	3 (25.0%)	0 (0%)	12
Park et al. [54]	2009	276	342	Mx. first molar (140) Mx. second molar (46) Md. first molar (118) Md. second molar (38)	70.2%	4.6±2.7y (mean) 10y	In periodontally diseased molars, bone support of >50% at the time of root resection was crucial. Failure more likely if the initial resection was due to root fracture and not periodontal disease.	51 (50%)	9 (8.8%)	19 (18.6%)	11 (10.8%)	12 (11.8%)	102
Derks et al. [52]	2018	69	90	Mx. first molar (22) Mx. second molar (7) Md. first molar (30) Md. second molar (23) Md. third molar (3) *Md. first molar (5) underwent root separation (hemisectioning)	98.9% (5y) 90.6% (10y) 68.9% (15y) 43.6% (20y) 26.7% (Mx molars, 20y) 76.8% (Md. molars, 20y)	14.7 ± 6.8y (mean) 4-30y	Mandibular molars had significantly lower risks of loss than maxillary molars (p=0.033). Cumulative survival rates drop from 90.6% to almost 80% from 10 to 20 years after root resection. 30/90 molars were extracted during a mean f/u of 14.7 years.	15 (50%)	8 (26.7%)	0 (0%)	5 (16.7%)	2 (6.7%)	30
Alassadi et al. [53]	2019	85	85	Mx. first molar (54) Mx. second molar (15) Md. first molar (14) Md. second molar (2)	98% (<1y) 79.6% (1-2y) 69.7%) (2-3) 56.8% (3-4y) 42.1% (8-12y) 18.4% (>12y)	5±4.3y (mean) 1-16.8y	Mean survival time of 9.1 years. >50% of teeth remained functional after nine years. 81.5% of all failures (n=31) within four years after treatment.	9 (23.7%)	2 (5.3%)	15 (39.5%)	10 (26.3%)	2 (5.3%) *Could not be determined.	38
Jepsen et al. [2]	2020	11	15	Mx. first molar (9) Mx. second molar (6)	100%	4.9y mean 3-7y	Vital root resection. No root canal treatment done. Pulpotomy followed by root amputation. All teeth had reduced probing depths, improved furcation status, no mobility, and stable clinical attachment.	0 (0%)	0 (0%)	0 (0%)	0 (0%)	0 (0%)	0
Ciardo et al. [16]	2024	5	7	Mx. molars (7)	100%	2.24 ± 0.45y (mean) 1.30-2.60y	Vital root resection with radicular retrograde partial pulpotomy. Seven vital maxillary molars treated using MTA. All had PPD ≥6 mm and class II+ furcation involvement. All remained in situ and clinically/ radiographically stable. Mean PPD improved from 4.02 ± 0.85 mm to 2.62 ± 0.42 mm. All remained thermally responsive.	0 (0%)	0 (0%)	0 (0%)	0 (0%)	0 (0%)	0
Oh et al. [58]	2025	60	60	Mx. molars (47) Md. molars (13)	65% overall 58% survival probability at 5y	Surviving teeth: 1 10.9y Extracted teeth: 2.9 ± 4y (mean) 0.2 13.4y	Retrospective cohort study. The failure rate was 35%, with 21 teeth extracted. 5y survival probability was 58%. Localized periodontal lesions had the highest prognosis. Carious/endodontic lesions had lower survival than periodontal or endodontic periodontal lesions. No complete coverage crown and most posterior tooth position were linked to poor survival.	3 (14.3%) Bone loss	0 (0%) Not reported as extraction cause	12 (57%)	4 (19%)	2 (9.5%) Persistent pain	21
Chen et al. [17]	2025	3	3	Mx. molars (3)	100%	1.58-2.67y	Three maxillary molars with class III furcation involvement were treated. All maintained pulp vitality during follow-up. Probing depths and mobility improved. Bone levels around remaining roots were maintained. No periapical pathology or adverse symptoms reported.	0 (0%)	0 (0%)	0 (0%)	0 (0%)	0 (0%)	0

Broader evidence on periodontally treated molars similarly emphasizes the importance of maintenance care and identifies furcation involvement, mobility, deep probing depths, smoking, diabetes, and poor compliance as risk factors for molar loss [59]. Mechanical complications are also relevant, as root amputation may reduce fracture resistance in splinted posterior units, particularly when furcation involvement is present [56]. These findings align with earlier studies showing that root resection can be clinically useful, but its long-term success depends on careful patient selection and long-term supportive periodontal care.

More recent case reports, published in 2026, continue to demonstrate the clinical versatility of root resection and hemisection in managing localized pathology while preserving natural dentition. Krsoum et al. (2026) reported successful management of a Grade 3 endodontic-periodontal lesion using palatal root resection combined with regenerative therapy, resulting in reduced probing depths, decreased mobility, resolution of infection, and radiographic healing after one year of follow-up [60].

Similarly, Ghani and Dhingra (2026) described successful hemisection of adjacent mandibular first and second molars affected by extensive carious destruction and localized periodontal involvement [61]. Clinical and radiographic follow-up demonstrated satisfactory healing, reduction in periodontal pocket depth, restoration of functional occlusion, absence of mobility, and maintenance of periodontal health following hemisection and prosthetic rehabilitation [61].

While Sokratous et al. (2026) presented four cases of root resection procedures to treat cases of vertical root fractures in lower molars [62]. All cases had a full coverage indirect restoration placed with long-term clinical and radiographic follow-up up to 10 years postoperatively, showing normal periapical and periodontal bone levels and probing depths, with absence of significant mobility, caries, or root fracture [62].

Discussion

Survival rates in the first 10 years ranged widely from 42.1% to 93.0%, which can be attributed to many factors. Case selection and surgical technique are often attributed to have the most important role in the prognosis of root resective treatment [5,21,32,49]. Others have implicated that differences in outcome definitions and follow-up periods also play a role in the varied survival rates [22]. This would be logical as many of the studies described in Table 2 had varied follow-up periods where some studies included the mean period, some included only the maximal period, and some included a broad range for their follow-up period.

Furthermore, studies with more descriptive follow-up periods, like those by Derks et al. (2018) and Alassadi et al. (2020), demonstrated a non-linear survival rate in root-resected molars [52,53]. Other authors have suggested that stricter inclusion criteria and a maintenance program could influence survival rates [54].

The anatomical differences between maxillary and mandibular molars and their respective roots could inherently play a role in how long a tooth stays functional before extraction is necessary. It would seem logical, as different teeth and their accompanying roots provide varying levels of attachment area. Despite this, and much like the lack of year-by-year information pertaining to the follow-up period, the inclusion of the specific root that was amputated is seldom included, and studies have yet to directly link the specific root amputated with its influence on survival rates.

Notably so, a study by Park et al. (2009) concluded that tooth-related factors had no significance on the survival rate after root resection [54]. An interesting trend noted in studies by Park et al. (2009) and Langer et al. (1981) is that maxillary molars were more prone to periodontal complications (P = 0.0489) while mandibular molars were more prone to root fractures (P = 0.0192) and dental caries (P = 0.0117) after root resective treatment [54,55]. However, this trend was not always readily apparent in other retrospective studies. While Langer et. al (1981) noted root fractures being the most common complication that leads to failure after root resective therapy, studies by Park et. al (2009) and Derks et al. (2018) found that periodontal complications were more prevalent in causing failure. Park et al. (2009) also noted that the reason for resecting a molar also played a role in its survival rate, as molars resected due to periodontal disease had a higher survival rate than molars resected due to fractures, endodontic problems, and dental caries (P = 0.0097) [54]. This information however is seldom included in the various studies contained in Table 2.

Moreover, there were inconsistencies in survival rates when comparing maxillary and mandibular molars between studies. Derks et al. (2018) noted a significantly lower risk of loss in mandibular molars compared to maxillary molars, while Langer et al. (1981) contrarily noted mandibular molars being two times more likely to fail than maxillary molars [52,55].

Loss of natural tooth structure during endodontic treatment, usage of posts or cores, and parafunctional habits have also been attributed to increasing susceptibility to root fractures [52]. The prosthetic role of a tooth after root resection is also important, as tooth mobility is more common when resected teeth are used as abutments [49]. To manage tooth mobility, some clinicians may opt to splint teeth together. However, one study found that intracoronally splinted root-resected teeth had significantly lowered fracture resistance [56].

Contrastingly, Park et al. (2009) found that tooth-related and patient-related factors had no statistically significant impact on ten-year survival rates after root resective therapy [54]. Tooth-related factors included the location of resected molars and roots, type of prosthetic abutment, opposing dentition, and postoperative prosthesis [54]. Patient-related factors included gender, age, smoking, and the remaining dentition [54]. Bone support for the remaining roots at the time of surgery had a significant effect (P = 0.0269) on survival rate, with remaining roots having more than 50% bone support being an important threshold [54]. Thus, having at least 50% bone support is an important factor when determining the candidacy of teeth for root resection therapy.

Many other factors play a role in survival rates, including case selection (furcation classification, prognosis, and candidacy for resection); surgical technique (endodontic, periodontal, and reconstructive dentistry); outcome definitions; follow-up period; the tooth or root that was resected; treatment indication (periodontal disease, obstructed root canal, root fracture, perforations…etc); post-treatment role or prosthesis (abutments, splints, and crowns); parafunctional habits; amount of tooth structure lost during endodontic treatment; amount of bone support for remaining roots prior to treatment; usage of posts and cores following endodontic treatment; and other treatment provided, such as supportive periodontal therapy or guided tissue regeneration procedures [5,21,32,49-54].

Taken together, these findings emphasize that root resection should be reserved for carefully selected cases, as periodontal breakdown, endodontic complications, root fracture, caries, inadequate remaining bone support, poor maintenance compliance, and unfavourable restorative or occlusal conditions may negatively influence prognosis [5,21,32,49-56]. These limitations are particularly important because root resection requires coordinated endodontic, periodontal, surgical, and restorative planning, and failure at any stage of treatment may compromise the long-term function of the retained tooth [4,5,21,32,49].

Treatment alternatives

While root resective therapy theoretically represents a viable means to correct furcation defects, its technique sensitivity and unclear long-term predictability make it a relatively unpopular technique. Clinicians often opt to pursue other modalities of treatment. For class II or III furcation-involved molars, this includes open flap debridement, guided tissue regeneration, tunnel preparation, and extraction and implant replacement [4,20].

While implants may be more expensive than root resective treatment, a previous study has indicated implant treatment to be less prone to complications [63]. In a retrospective study comparing hemisectioned mandibular molars to mandibular implants, it was noted that, at five-year follow-up, 68% of patients who received hemisections were free of complications compared to 89% of patients who received implant treatment [63]. More specifically, patients who received hemisections had a greater incidence of overall complications (P = 0.027) as well as nonsalvageable complications (P = 0.013) [63].

However, more recent literature has challenged the assumption that extraction and implant placement should be considered the default treatment for periodontally compromised teeth. In a systematic review comparing tooth preservation with extraction and implant placement in periodontally compromised patients, Sarafidou et al. (2022) found that both approaches demonstrated high survival rates after at least five years of follow-up [64]. Survival ranged from 81.8% to 100% in the tooth-retention group and from 94.8% to 100% in the implant group, suggesting that tooth preservation may remain a viable option when appropriate periodontal therapy, regeneration, and maintenance are provided. Similarly, Afrashtehfar et al. (2024) concluded that both implant placement and tooth preservation can demonstrate favourable long-term outcomes, but emphasized that treatment decisions should consider tooth restorability, periodontal stability, patient-specific risk factors, and long-term maintenance rather than survival rates alone [65].

This discrepancy could be explained by differences in technique sensitivity as well as the clinician’s familiarity with either procedure. The hemisectioned treatment was also done by different clinicians, as general dentists performed the endodontic treatment and periodontists performed the resective treatment [63]. While various procedures may be effective, the cost-benefit ratio will need to be weighed by clinicians and patients.

A commonly overlooked cost associated with implant placement is the biological cost. That is, once a tooth is extracted, it cannot be replaced with a natural tooth. Considering that no treatment in dentistry is permanent, eventually, all implant treatments are destined to fail. With implant failure often comes severe bone loss and morbidity, making it more difficult to replace the edentulous space, especially with another implant. Considering that the average lifespan of humans is increasing with time, it may make sense to delay implant treatment to later in life. Contrastingly, if a tooth is saved with endodontic or periodontic therapy, including root resection, it can be replaced with an implant if issues arise, but not the other way around. Thus, there is a case for root resection therapy to preserve natural tooth structure as long as possible, prior to tooth replacement options, as a last resort.

Novel applications

Recent studies have identified novel applications for root resection and hemi-section procedures, including the orthodontic management of missing permanent premolars; managing peri-operative endodontic complications; and managing anterior teeth with fusion or macrodontia [66-69].

A randomized controlled trial of patients congenitally missing lower second premolars evaluated hemisection and removal of the distal portion of mandibular primary second molars to facilitate mesial drift of the permanent first molars and favourable orthodontic treatment [66]. The mesial portion of the tooth was later extracted, on average 11.54 months following hemisection, with many cases (48% with pulp capping, and 33% without) resulting in reparative tertiary dentin formation around the pulp [66]. The majority of patients were asymptomatic, with three of the 21 reporting symptoms at follow-up [66]. The trial highlights the reparative capacity of primary teeth and the potential for interceptive hemisection in the mixed dentition, with coordinated orthodontic treatment.

A recent case series by Qaiser et al. presents a novel use of hemisection to treat symptomatic cases of attempted RCT that resulted in irretrievable instrument separation in one root of mandibular molars [67]. In both cases, the affected root was hemisectioned and extracted, with the remaining tooth portion protected with a full-coverage indirect restoration [67]. Six-month follow-up showed radiographic signs of healing, including bone formation in the extraction site and no evidence of apical periodontitis [67]. This case series highlights the potential for hemisection or root resection in the management of perioperative complications of RCT, particularly when patients present symptomatically at follow-up visits after instrument separation, perforation, ledging, or treatment failure at one root.

Two case reports have identified therapeutic contexts for hemisection in anterior teeth [68,69]. A case of an eight-year-old patient with gingival recession at a lower central incisor fused to a supernumerary tooth, with separate roots and pulp complex, was successfully treated with hemisection, extraction of the supernumerary tooth, coronally advanced flap, and an indirect restoration on the remaining tooth, without RCT [68]. Follow-up at two years and eight months demonstrated healing, pulp vitality without sensitivity, and stable gingival contour [68].

The second case was of an 11-year-old with upper central incisor macrodontia, associated with a variant of the Ekman-Westborg and Julin trait [69]. The two central incisors were hemisectioned using a surgical guide to remove the mesial portions, with partial pulpotomy used to treat the pulp exposures and orthodontic treatment to close the resultant diastema [69]. 39-month follow-up revealed normal pulp vitality, probing depths and no radiographic signs of pathology on CBCT imaging [69]. These two cases highlight the potential for modified hemisection techniques to treat dental anomalies in anterior teeth, particularly in the mixed dentition and in combination with orthodontic treatment, though they should be investigated further as more long-term follow-up data is required.

## Conclusions

Root resective therapy offers clinicians an alternative treatment modality when the preservation of natural, multi-rooted teeth is preferred. Here we reviewed the literature on root resective techniques and studies assessing their success rates. Though the prognosis varied, we conclude they are moderately predictable and still have a place in modern dentistry. The predictability of root resective therapy may be improved by optimizing case selection, improving surgical proficiency, and utilizing modern biomaterials to aid in recovery and rehabilitation. Current studies are limited in sample size, length of follow-up, controlling for confounding variables, and measuring patient-related outcomes, like oral health-related quality of life. Long-term standardized randomized controlled trials are needed to address these limitations and further validate the efficacy and success of root resective therapy.
